# Effect of Excess Atomic Volume on Crack Evolution in a Deformed Iron Single Crystal

**DOI:** 10.3390/ma14206124

**Published:** 2021-10-15

**Authors:** Dmitrij S. Kryzhevich, Aleksandr V. Korchuganov, Konstantin P. Zolnikov

**Affiliations:** Institute of Strength Physics and Materials Science of Siberian Branch of Russian Academy of Sciences, Akademicheskii 2/4, 634021 Tomsk, Russia; avkor@ispms.ru (A.V.K.); kost@ispms.ru (K.P.Z.)

**Keywords:** molecular dynamics, crack, crack healing, excess atomic volume, iron, uniaxial tension, shear

## Abstract

This paper presents a molecular dynamics study of how the localization and transfer of excess atomic volume by structural defects affects the evolution and self-healing of nanosized cracks in bcc iron single crystals under different mechanical loading conditions at room temperature. It is shown that deformation is initially accompanied by a local growth of the atomic volume at the crack tips. The crack growth behavior depends on whether the excess atomic volume can be transferred by structural defects from the crack tips to the free surface or other interfaces. If an edge crack is oriented with respect to the loading direction so that dislocations are not emitted from its tip or only twins are emitted, then the sample undergoes a brittle-ductile fracture. The transfer of the excess atomic volume by dislocations from the crack tips prevents the opening of edge cracks and is an effective healing mechanism for nanocracks in a mechanically loaded material.

## 1. Introduction

Body-centered cubic materials such as iron are widely used as structural materials in various industries. To improve the performance of structures and products, it is very important to study the initiation and evolution of fractures in materials at the atomic scale. Fracture processes are associated with crack initiation and growth in materials subjected to various complex combinations of mechanical, thermal and chemical loads. Despite the fact that fractures can occur at different scale levels, they always originate at the atomic scale as a result of local structural transformations caused by stress redistributions within the material [1,2,3,4]. The development of fractures at the atomic scale is accompanied by the breaking of atomic bonds and emission of dislocations/twins from the tips of nanocracks. As a result, the fracture behavior of the material is largely determined by the local atomic environment in the crack region, in particular, the atomic structure, lattice orientation, and the presence of structural defects and interfaces.

Iron single crystals with low silicon content are a good model material for the experimental study of brittle-ductile fractures [5,6,7,8]. The elastic constants in this material differ insignificantly from the same parameters in pure bcc iron. Therefore, the results of fracture simulation in single crystals with the bcc lattice of pure Fe can be correctly compared with experimental data for iron single crystals with low Si content [9,10,11]. The cracking patterns in metallic materials strongly depend on the evolution of the structure around the crack tip. For example, the transition from brittle to ductile crack growth in a material is often associated with the generation of dislocations in the crack tip region [2] The formation of twins at the crack tip in a bcc crystal was shown experimentally in [12]. A detailed study of twinning and recrystallization at the crack tip at low temperatures in single-crystal and nanocrystalline bcc iron can be found in [13,14]. The cracking behavior of iron samples depends on various external and internal parameters, such as temperature [2], strain rate [15], type of load [7,16], and crack orientation [6,7].

Loading can lead to crack healing and thus enhance the fracture resistance of the material. This phenomenon is of great theoretical and practical interest, since knowledge of healing mechanisms could suggest ways to extend the life of materials and achieve significant economic benefits [17,18,19]. The ability of cracks to close has been found in a variety of materials: polymers, ceramics, graphene, metals and alloys [20,21,22]. The research results showed that cracks in bcc iron can also close to a certain extent during heating and compressive loading [23,24]. It was suggested [25] that the thermal or mechanical treatment promotes the reduction of surface energy and healing of microcracks. Molecular dynamics simulations of the behavior of Fe samples with microcracks at increasing temperature showed that microcrack healing is due to the diffusion of Fe atoms into the crack region, rather than due to thermal stresses [24]. Additional compression of heated samples enhances the crack healing process.

The transition from brittle to ductile crack growth and the crack healing ability, accompanied by the generation of dislocations, twinning and local structural transformations, including recrystallization and phase transitions, are caused by a significant redistribution of atomic density. These phenomena are always related to the formation of local regions with excess atomic volume and the transfer of this volume by various defects during loading or relaxation of the loaded material [26,27,28].

The available experimental data indicate an important role of the excess atomic volume in the initiation of plasticity in metals [29,30]. It was shown by high-resolution TEM that a 5% local increase in interatomic distances precedes the nucleation of a partial dislocation in a tensile gold nanocrystal [29]. An increase in the atomic volume is necessary for the homogeneous melting of metals with a perfect bcc lattice, which was established for a vanadium crystal by molecular dynamics simulations [30]. The structural transformation processes at the crack tip and the emission of dislocations/twins from this region are also directly related to a significant redistribution of atomic volume.

In the last decade, the above non-linear processes have been increasingly investigated by molecular dynamics simulations, along with experimental observations. This approach provides detailed information on the effect of the crystallographic orientation of the sample, loading schemes, interfaces, impurities, and structural defects, by directly taking into account the discrete structure at the crack tip. It allows one to most effectively study the relationships between the redistribution of the excess atomic volume and structural evolution during material deformation.

The relationships between the fracture mechanisms, including crack healing, and the redistribution of the excess atomic volume due to the initiation and growth of various structural defects have not yet been fully explored.

Here we examine how the redistribution of the excess atomic volume in the crack tip region affects the evolution and healing of cracks in a bcc iron single crystal under various loading conditions.

## 2. Materials and Methods

**Figure 1:**
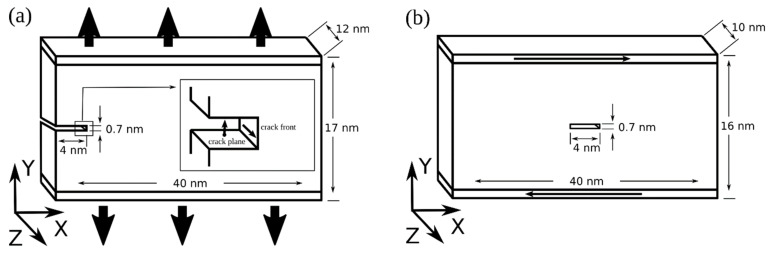
Loading scheme for samples with an edge (**a**) and a central (**b**) crack.

Crack evolution in iron was investigated by molecular dynamics simulations using the LAMMPS code [31]. The interatomic interaction was described by a many-body potential calculated with the embedded atom method in the Finnis‒Sinclair approximation [32]. The investigation was performed on iron single crystals with an edge and central incipient crack. Samples with an edge crack contained about 800,000 atoms, and samples with a central crack contained 550,000 atoms. The loading scheme is shown in Figure 1; the orientations of cracks and samples are given in the Table 1. The orientation of the incipient crack was denoted by the crack plane and crack front indices. Free surfaces were directed along the X axis. Periodic boundary conditions were imposed in the Z direction. The load on the planes was applied in the Y direction. Single crystals with an edge crack were stretched between grips displaced in the Y direction (soft boundary conditions) with a force sufficient to open the crack. The value of the tensile force was selected in such a way that the opening of incipient cracks in the samples began in 60–80 ps after loading. Shear in single crystals with a central crack was simulated by moving the rigid grips along the X axis at a constant velocity of 5 m/s in opposite directions. Samples with an edge crack had dimensions of 40 × 17 × 12 nm^3^, and samples with a central crack measured 40 × 16 × 10 nm^3^. The crack size was 4 × 0.7 × 10 nm^3^. The grip thickness was 0.7 nm. Before loading, the sample was relaxed using a Langevin thermostat for 5 ps at a temperature of 300 K. The integration step was equal to 1 fs.

The atomic volume was calculated by constructing Voronoi polyhedra. The magnitude of the atomic volume was given relative to an equilibrium value at a temperature of 300 K. Analysis of the simulation results and identification of local structural changes in the crystallites were carried out using the common neighbor analysis algorithm for each atom [33]. This algorithm was applied to identify fcc, bcc, and hcp lattices. In the case when the nearest neighbor structure of atoms could not be classified, the algorithm identified them as atoms with uncertain nearest neighbor symmetry. The structure of the simulated crystallites was visualized using the OVITO software [34].

For calculating the average volume at the tip of an edge crack, we constructed a cylinder with a radius of 3 to 4 nm, depending on the size of the region of structural changes. The cylinder axis coincided with the *Z*-axis direction. The volume of a central crack was calculated by summing up the volume of Voronoi cells of surface atoms.

## 3. Simulation Results and Discussion

**Figure 2:**
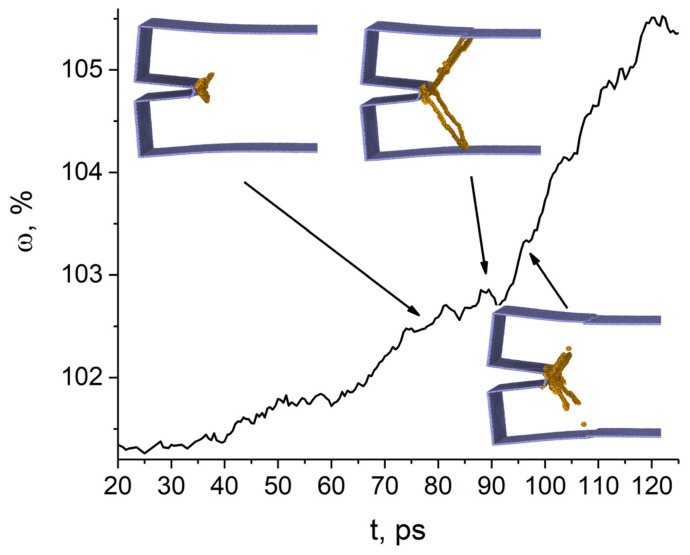
Time variation of atomic volume at the tip of the (01$\bar{1}$)[011] crack. Surface atoms are colored blue, dislocations are colored orange. Atoms with bcc nearest neighbor symmetry are not shown.

Our simulations showed that the formation of the excess atomic volume during loading is determined by the crack growth behavior and the emission of structural defects from the region around the tip of the crack. Edge cracks initially grow in width, which is uneven along the crack length. The closer to the free surface, the more the gap between the crack faces during loading. The atomic volume in the tip region does not change until the crack begins to open. As follows from the analysis of the simulation results, the faces of the (01$\bar{1}$)[011] crack almost do not move in the interval from 20 to 33 ps, and the atomic volume remains practically unchanged. With further loading in the range of 35–93 ps, the crack width increases, but with a slow increase in the atomic volume in this region accompanied by the appearance of pronounced peaks in the curve of Figure 2. The variation of atomic volume is caused by the emission of dislocations from the crack tip region. The dislocations glide on the {112} planes and partially carry away the excess atomic volume. As a result, its expansion rate is significantly reduced. These and subsequent dislocation emissions from the tip region are correlated with the appearance of relatively flat portions in the curve of the excess atomic volume versus time (Figure 2). The escape of dislocations to the loaded surface causes the formation of steps on it. According to the simulation results, the dislocation emission first slows down the growth of tensile stresses and then leads to their decrease. Changes in the excess atomic volume at the crack tip and in tensile stresses correlate well. Thus, the opening of the (01$\bar{1}$)[011] crack is suppressed due to the transfer of the excess atomic volume by dislocations from the crack tip to the loaded surfaces. Note that atomic bond breaking is possible only at a certain threshold value of the excess atomic volume. Our simulation results are in good agreement with the results of Ringdalen et al. [11]. They reported for an iron single crystal under mode I loading that edge dislocations are emitted on {112} planes from the tip of the (01$\bar{1}$)[011] crack. According to experimental data [7], an Fe-3 wt.% Si single crystal with the given crack orientation in mode I undergoes slow ductile fracture with dislocation emission on {112}<111> slip systems from the crack tip. It can be assumed that an increase in the size of the simulated sample and the loading time will lead to a ductile fracture, because the atomic volume at the crack tip increases during loading faster than it is carried away from it by dislocations.

**Figure 3:**
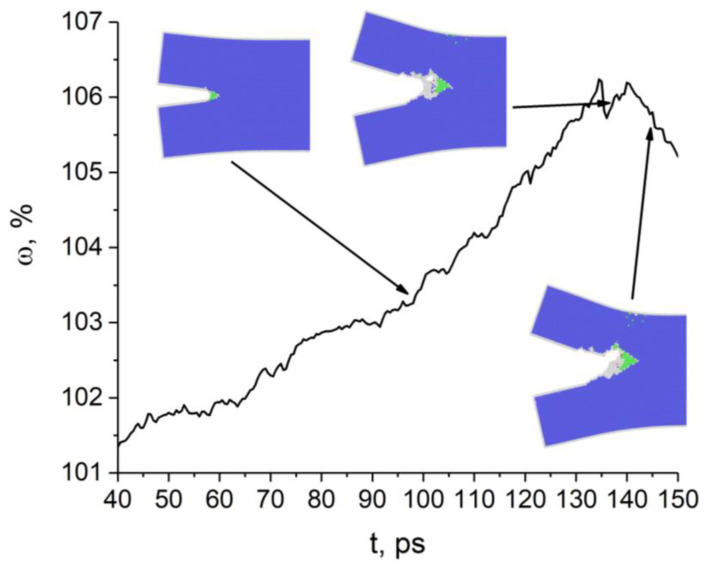
Time variation of atomic volume at the tip of the (010)[100] crack. Atoms with bcc, fcc, and uncertain nearest neighbor symmetry are colored blue, green and gray, respectively.

The change in the atomic volume at the tip of the (010)[001] crack also correlates well with the crack growth behavior at the initial loading stage until 93 ps (Figure 3). The crack does not open and the excess atomic volume does not increase where the curve slopes gently (intervals 45–65 ps and 75–90 ps). Later, however, the crack opened in a stick-slip manner. Unlike the previous case, structural defects are not emitted from the crack tip during loading. Areas with uncertain or the fcc structure are formed in the tip region. Atomic bond breaking occurs in the zone with uncertain structure, not with fcc. The crack opens in the [110] direction through the formation of a nanopore and its subsequent coalescence with the crack. The insets in Figure 3 show the nanopore at the time point 133 ps and at 145 ps when it coalesces with the crack.

Experimental data for iron single crystals with 3 wt.% Si with the same crack orientation revealed a strong effect of the sample size on the crack growth behavior in mode I [35]. The behavior of the crack in millimeter-sized samples is brittle, while in micrometer-sized samples it is ductile and accompanied by dislocation emission on {112} planes. The differences in the fracture behavior of the samples are explained by a reduced volume of the plastic zone in micrometer-sized samples [35].

**Figure 4:**
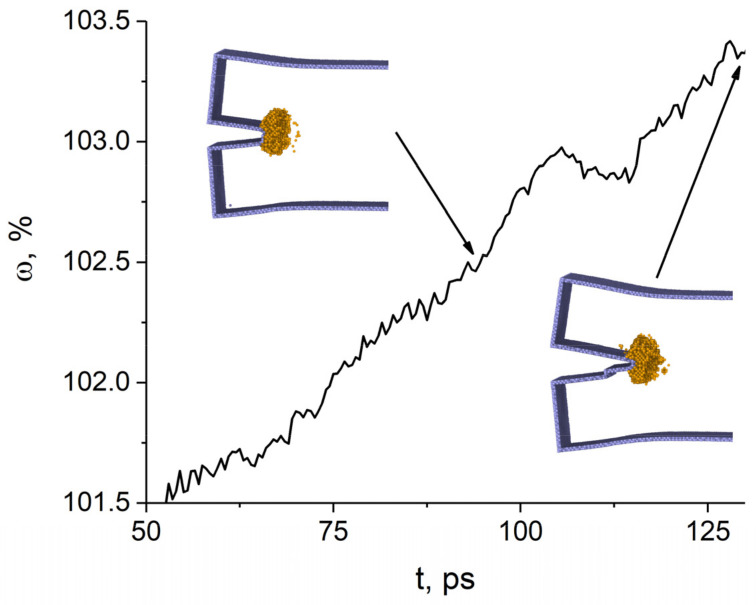
Time variation of atomic volume at the tip of the (110)[001] crack. Surface atoms and atoms with volume >105% are colored blue and orange, respectively. Bcc atoms with volume <105% are not shown.

**Figure 5:**
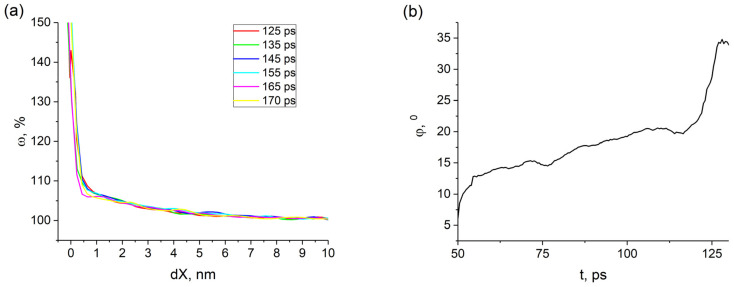
Distribution profile of atomic volume along the X axis relative to the crack tip of the (110)[001] crack for the corresponding points in time (**a**). Time variation of the crack opening angle (**b**).

The single crystal with the (110)[001] crack under tensile deformation undergoes brittle fracture in the (110) plane. At the time point 80 ps, a zone with the fcc structure begins to form at the crack tip. The bcc–fcc phase transformation in this zone, which is observed for some crack orientations, requires very high stresses. They usually arise in this area if no defects are emitted from the crack tip [11]. The excess atomic volume is strongly localized near the crack tip and increases during loading until 108 ps (Figure 4). As can be seen from Figure 4, the atomic volume in the tip region remains unchanged in the interval from 108 to 115 ps, because the crack does not open. However, the crack propagates into the sample in this interval. The zones with the fcc structure on both sides of the crack tip are preserved and slightly expand. The crack orientation and the loading scheme of the sample are such that atomic bonds preferably break in (110) planes. Note that the distribution profile of the excess atomic volume in the plane in front of the propagating crack along its entire path almost does not change (Figure 5a). Minor deviations in the distribution profiles in Figure 5a for different times are due to a number of factors, such as thermal fluctuations of atoms, an increase in the curvature of the crack faces and the crack opening angle (Figure 5b) during loading. The influence of these factors will increase with loading time and, as a result, brittle crack growth will change to ductile.

**Figure 6:**
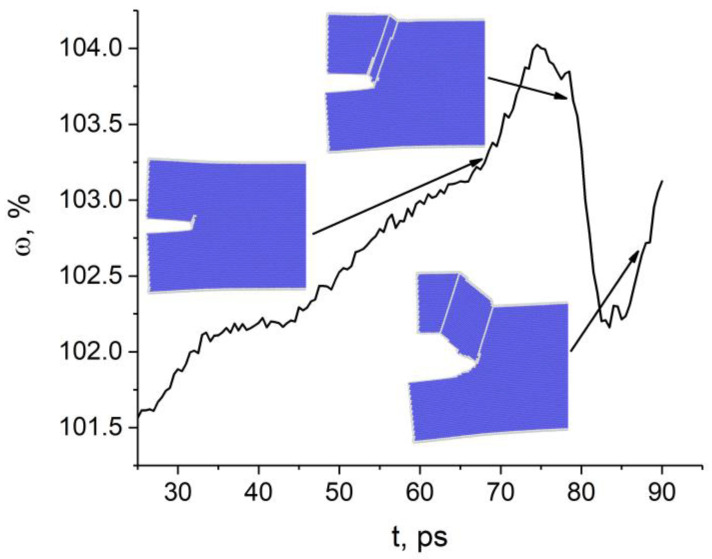
Time variation of atomic volume at the tip of the ($11\bar{2}$)[$\bar{1}$ 10] crack. Atoms with bcc and uncertain nearest neighbor symmetry are colored blue and gray, respectively.

The results of calculating the atomic volume variation at the tip of the ($11\bar{2}$)[$\bar{1}$10] crack are presented in Figure 6. Until 95 ps, the change in the atomic volume at the crack tip correlates well with the change in the width of the crack caused by tensile loads. At 55 ps, a twin forms near the crack tip and its evolution leads to a significant increase in the atomic volume in the range from 55 to 75 ps. Then the twin arrives at the surface and grows in thickness, which is accompanied by an abrupt decrease in the atomic volume at the crack tip. Analysis of atomic displacements shows that the atomic volume decreases due to the displacement of the entire region to the right of the twin boundary toward the center of the sample. Thus, a twin boundary is formed in the sample, which propagates from the crack tip towards the sample surface. Starting from 80 ps, the atomic volume begins to grow again and the crack opens. The twin boundary propagates together with the crack tip.

**Figure 7:**
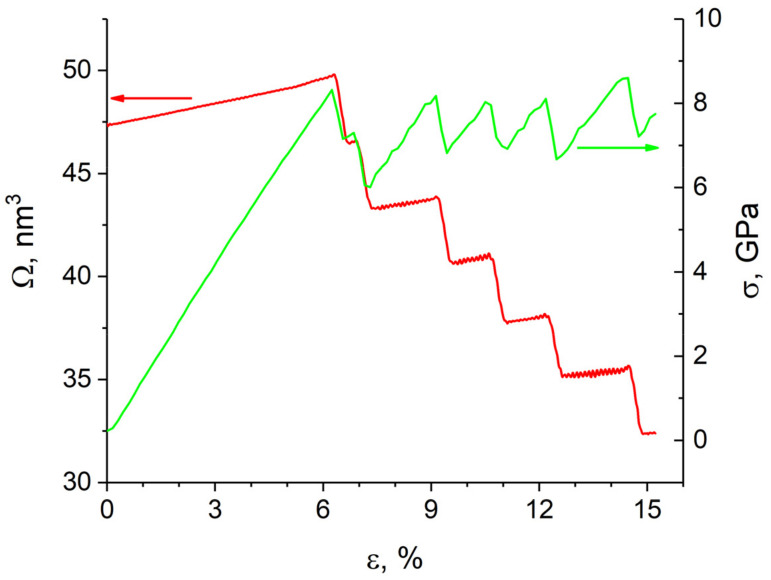
Variation of stress intensity of the sample (green curve) and crack volume (red curve) versus strain degree for sample with (11$\bar{2}$)[$\bar{1}$ 10] crack. Axis corresponding to each curve is pointed by an arrow.

**Figure 8:**
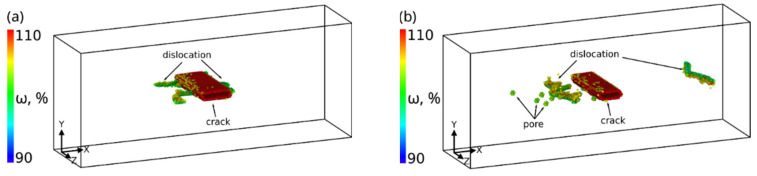
Defect structure at 6.3% (**a**) and 12.4% (**b**) shear deformation for sample with (11$\bar{2}$)[$\bar{1}$ 10] crack. Bcc atoms with volume <105% are not shown.

The simulation results showed that shear loading of a sample with the (11$\bar{2}$)[$\bar{1}$10] crack up to 6.5% strain is elastic (Figure 7). The rectangular incipient crack becomes diamond-shaped during loading, and upon reaching the yield point an edge dislocation is emitted from its tip (Figure 8a). It is seen from Figure 7 that as a result of the dislocation emission the volume of the crack decreases and the internal stresses relax. According to TEM results [36], a crack in bcc Fe is completely healed by heating the sample to 1073 K. It was assumed in [25] that thermal or mechanical treatment of the material provides a driving force for minimizing the surface energy and for crack healing. Note that there is a periodic emission of dislocations from the crack tip during loading when the maximum stresses are reached. The emission period is determined by the grip velocity and the lattice period in the tensile direction. All generated dislocations glide on the (111) atomic plane, which is the most favorable for dislocation glide in the bcc structure. Upon reaching the free surface, they form a growing step on it. The dislocations leave behind a small amount of vacancies in the slip plane (Figure 8b). For a complete healing of the (11$\bar{2}$)[$\bar{1}$10] crack of the given size, the shear strain must be equal to ~30%.

**Figure 9:**
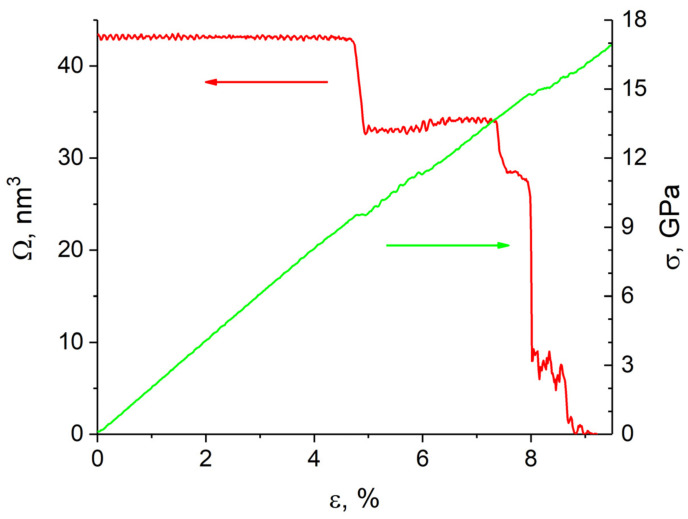
Variation of stress intensity of the sample (green curve) and crack volume (red curve) versus strain degree for sample with (01$\bar{1}$)[011] crack. Axis corresponding to each curve is pointed by an arrow.

**Figure 10:**
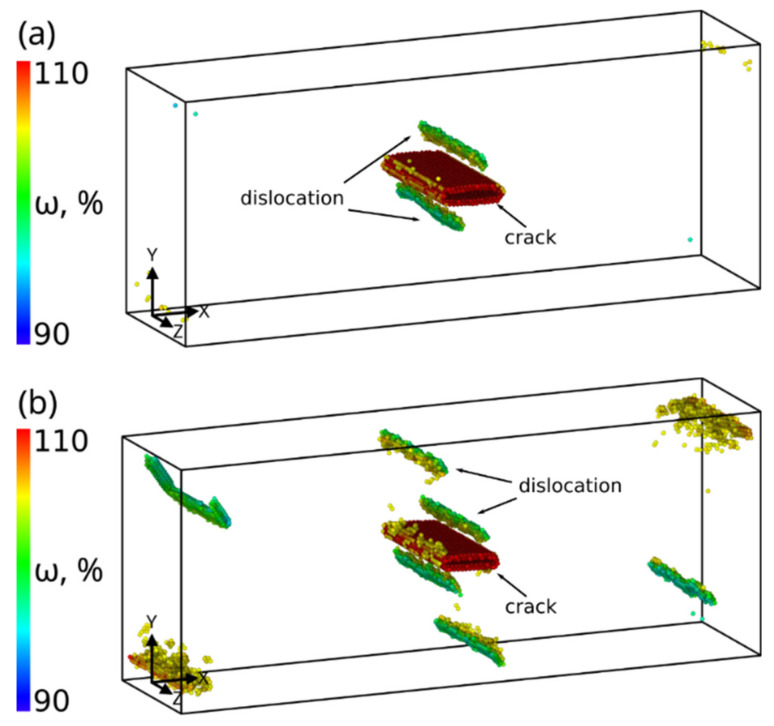
Defect structure at 4.5% (**a**) and 7.4% (**b**) shear deformation for sample with (01$\bar{1}$)[011] crack. Bcc atoms with volume <105% are not shown.

A (01$\bar{1}$)[011] crack also heals through dislocation emission from its tips under shear loading. In this case, each edge dislocation reduces the crack volume by almost two times more than in the previous case (Figure 9). As can be seen from the figure, the change in the nanocrack volume correlates well with the internal stress variation in the sample. Stresses in the single crystal of the given orientation are concentrated, in addition to crack tips, in the corners of the sample where the grips are attached to the free surface. Such stress concentrations are due to incompatible deformations between the grips, the deformed region of the sample, and the free surface. During loading, edge dislocations are also emitted from the stress concentration zones (Figure 10). The dislocations glide on {111} planes and leave no vacancies behind. The thickness of the healed crack decreases after each dislocation emission event; its faces close and become curved at the tips. Since the grips were simulated by rigid non-deformable regions, dislocations could not arrive at the surface and remained in the bulk of the material. With the given sample orientation and loading scheme, the internal stresses increased during the entire loading period (Figure 9).

## 4. Conclusions

This study of fracture of iron single crystals with different types and orientations of incipient cracks showed that the formation and evolution of the excess atomic volume at the crack tips determine the deformation behavior of the material.

For a sample with the (01$\bar{1}$)[011] edge crack under uniaxial tension, the excess atomic volume is constantly carried away from the crack tip by dislocations, which reduces the stresses in this region and prevents crack propagation into the material bulk. Ductile fracture in this region is also prevented by the small size of the plasticity zone, determined by the nanoscale dimensions of the simulated single crystal.

The uniaxial tension of a sample with the (010)[001] crack leads to a rapid increase in the excess atomic volume at the crack tip. Structural defects are not emitted from the tip and, therefore, the internal stresses increase significantly and the fcc structure is formed in this region. With further loading, the crack grows in a ductile manner.

The growth of the (110)[001] crack in the simulated sample is brittle and occurs in the (110) plane. Such behavior is caused by the localization of the excess volume, high stresses, and the absence of structure relaxation mechanisms at the crack tip. The high stresses and the lack of emission of defects lead to fcc structure formation in the tip region. With further expansion of the excess atomic volume, the fracture mode changes to a brittle one, which is based on direct and reverse bcc–fcc–bcc transformations at the tip.

In a tensile sample with the (11$\bar{2}$)[$\bar{1}$10] crack, the excess volume at the crack tip first increases, but then abruptly decreases due to twin emission. A twin boundary forms at the crack tip. As a result, a part of the sample is reoriented and it undergoes ductile fracture, despite the relatively small excess atomic volume in this region.

It is shown that the transfer of the excess atomic volume by dislocations from the vicinity of nanoscale cracks leads to crack healing under shear loading. The size of the (11$\bar{2}$)[$\bar{1}$10] and (01$\bar{1}$)[011] nanoscale cracks in the bulk of the samples reduced abruptly with each emission of edge dislocations. The dislocation emission was periodic, depending on the shear rate and the lattice period, and continued until the complete healing of the cracks.

The simulations performed are in good agreement with the experimental and numerical results on the fracture of bcc Fe single crystals.

## Figures and Tables

**Table 1:** Simulated samples.

Crack Location	Crack Orientation	Sample Orientation
Edge	(01$\bar{1}$)[011]	X [100], Y [01$\bar{1}$], Z [011]
Edge	(010)[001]	X [100], Y [010], Z [001]
Edge	(110)[001]	X [1$\bar{1}$0], Y [110], Z [001]
Edge	(11$\bar{2}$)[$\bar{1}$10]	X [111], Y [11$\bar{2}$], Z [$\bar{1}$10]
Central	(11$\bar{2}$)[$\bar{1}$10]	X [111], Y [11$\bar{2}$], Z [$\bar{1}$10]
Central	(01$\bar{1}$)[011]	X [100], Y [01$\bar{1}$], Z [011]

## Data Availability

Data sharing not applicable.
